# Supplementary material for: Genome-Wide Survey for Microdeletions or -Duplications in 155 Patients with Lower Urinary Tract Obstructions (LUTO)
Source: Genes (Basel). 2021 Sep 20;12(9):1449. doi: 10.3390/genes12091449 (PMC8468665; doi:10.3390/genes12091449)
Supplement: Supplementary file 1 [file genes-12-01449-s001.zip › 2021-07-31 LUTO CNV Analysis supplemental figure.pdf]

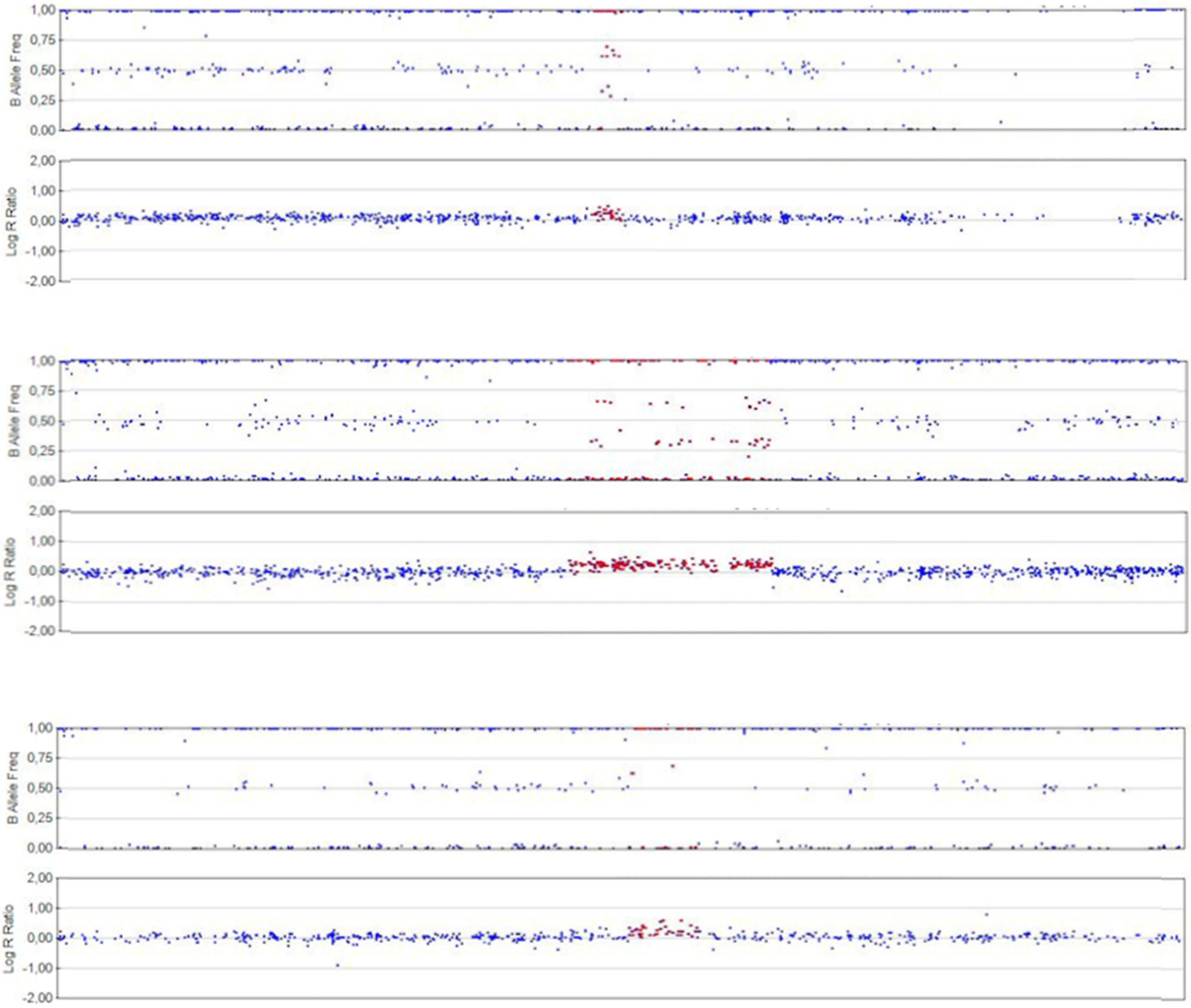

(a)

(b)

(c)

Supplemental Figure 1. Illumina GSA 1.0 hybridization plot of microduplications at 1p36.21, 5q23.2 and 10q23.31 in patients with LUTO**.** Log R ratio (bottom) represents a measure of the signal intensity for each marker and B allele frequency (top) denotes an allelic intensity ratio for each SNP. **(a)** dup1p36.21 detected in patient 100009 with LUTO; **(b)** dup5q23.2 detected in patient 100243 with PUV; **(c)** dup10q23.31 detected in patient 100295 with PUV.
